# New Appliances and Things Medical

**Published:** 1898-01-29

**Authors:** 


### NEW APPLIANCES AND THINGS MEDICAL.

**[We shall be glad to receive, at our Office, 28 & 29, Southampton Street, Strand, London, W.C., from the manufacturers, specimens of all new preparations and appliances which may be brought out from time to time.]**

### WELCH'S GRAPE JUICE.

(THE WELCH GRAPE JUICE COMPANY, WATKINS, N.Y.; TAYLOR AND WEAVER, 12, LANCELOT'S HEY, LIVERPOOL).

Welch's grape juice is a non-intoxicating beverage, and is said to be prepared from the finest Concord (U.S.A.) grapes. In appearance and flavour it resembles an unfermented grape juice of a red wine character, and this view is corroborated by analysis, which agrees with the composition of a non-intoxicating grape juice. The sample examined contained 22 per cent. extractures, 0·9 per cent. acidity, expressed as tartaric acid, 0·3 per cent. mineral constituents, but no alcohol. The juice was somewhat cloudy, but it soon became brilliant on being slightly warmed.

### VALSŌL PREPARATIONS.

(WILLIAM POPPELREUTER, 54, PORTLAND STREET, MANCHESTER.)

Valsōl or oxygenated petroleum (Klever) has recently been adapted as a vehicle for many internal and external medicaments which have hitherto offered many difficulties to solution in the ordinary pharmaceutical bases. Valsōl, which is a petroleum containing a considerable percentage of absorbed oxygen, has apparently properties which do not belong to the more usual form of vaseline. Its chief virtue appears to consist in its ready and immediate absorption by the skin, whether broken or intact, the rationale being that valsōl with the secretion of the skin at once forms an emulsion which readily makes its way into the lymphatics and capillaries of the underlying tissues. If experience proves that it is absorbed as rapidly as is claimed for it, it is open to question whether in many cases inunction with this new mineral oil may not entirely supersede hypodermic injection. A large number of medicaments in solution in valsōl have been put before us, and, as far as we have examined, the percentage of the drug is as stated by the manufacturers. The iodine valsōl (6 per cent. and 10 per cent.) is a particularly useful preparation, as it does not stain the skin permanently, but only for a few minutes. Iodoform, creasote, ichthyol, salicylic acid, and hyd. biniodide are at present perhaps the most useful preparations prepared on the new process with the oxygenated petroleum. We intend to make careful clinical trials of the valsōl preparations, and we recommend our readers to do the same.

### REFINED BEEF SUET—"ATORA" BRAND.

(HUGON AND CO., PENDLETON, MANCHESTER.)

We have on previous occasions made mention of the above refined suet. We again call attention to some of its more obvious advantages as compared to ordinary suet or lard. In the first place, it is sent out by the manufacturers in a most convenient form for storage in the larder, *i.e.*, in 1 lb. packets carefully wrapped up. In this form it can be kept without injury or development of rancidity for two months—the sample before us has been this length of time in our office under observation. Further, as regards time required for chopping, it compares favourably with ordinary suet, and the price is 8d. per lb. We recommend it to the notice of our readers, believing that in Hugon's suet they will find a pure and most satisfactory article.

### VI-COCOA.

(DR. TIBBLES' VI-COCOA, LIMITED, LONDON.)

This is a preparation of cocoa to which something is said to have been added which is claimed to impart to it great sustaining powers, and the question has not unnaturally been asked whether it is to be looked upon as a drug or a food. It is said to contain the active principle of the kola nut, and if this substance is desired we see no possible harm in taking it in combination with cocoa. The results of the analytical examination of Vi-Cocoa are in most respects the same as those obtained from the various preparations of cocoa bean. If the proprietors would take the necessary steps to inform the public as to the extent to which the special substances on which its alleged virtues are said to depend are added to the cocoa, which is the basis of the preparation, the medical profession would probably feel more satisfaction in sanctioning its use than they can do while it remains a secret preparation. As a cocoa it is very good, and we fancy it is mainly as a cocoa that it is used.

